# Narratives of children who hear voices: nature, characteristics and stories

**DOI:** 10.1590/0034-7167-2024-0314

**Published:** 2025-11-21

**Authors:** Clarissa de Souza Cardoso, Valéria Cristina Christello Coimbra, Luciane Prado Kantorski, Milena Hohmann Antonacci, Danton Matheus de Souza, Liamara Denise Ubessi

**Affiliations:** I Universidade Federal do Pampa. Uruguaiana, Rio Grande do Sul, Brazil; II Universidade Federal de Pelotas. Pelotas, Rio Grande do Sul, Brazil; III Universidade de São Paulo. São Paulo, São Paulo, Brazil

**Keywords:** Mental Health, Child Health, Social Support, Pediatric Nursing, Health Promotion., Salud Mental, Salud Infantil, Apoyo Social, Enfermería Pediátrica, Promoción de Salud.

## Abstract

**Objectives:**

to understand narratives of children who hear voices, through their nature, characteristics and stories.

**Methods:**

a narrative and qualitative study. Benjamin’s concepts of narrative and memory were used to analyze the contribution of four children who self-declared as voice hearers.

**Results:**

from narratives, three categories emerged: 1) Children’s multisensory experience: nature of experiences (all had voices, not all had visions or other sensations); 2) Hearing voices: an unusual beginning (narratives covered the month of beginning, time of day and activities carried out at the time); and 3) Voice characteristics: who are they? (the gender, age range, tone of voice, frequency and content of voices/visions were described).

**Final Considerations:**

it was observed, from narratives, that children experience voices, visions and sensations in a unique, personal and non-transferable manner, and the construction of dialogue in the group promoted acceptance and even a positive coexistence with the phenomenon.

## RESUMEN

**Objetivos:**

comprender las narrativas de los niños y niñas que escuchan voces, a través de su naturaleza, características e historias.

**Métodos:**

estudio narrativo con enfoque cualitativo. Se utilizaron los conceptos de narrativa y memoria de Benjamin para analizar la contribución de cuatro niños que se autodeclararon oyentes de voces.

**Resultados:**

de las narrativas emergieron tres categorías: 1) La experiencia multisensorial de los niños: naturaleza de las experiencias (todos tenían voces, no todos tenían visiones u otras sensaciones); 2) Escuchar voces: un comienzo inusual (las narraciones cubrieron el mes de inicio, la hora del día y las actividades realizadas en el momento); y 3) Características de las voces: ¿quiénes son? (se describió género, rango de edad, tono de voz, frecuencia y contenido de las voces/visiones).

**Consideraciones Finales:**

se observó, a partir de las narrativas, que los niños experimentan voces, visiones y sensaciones de forma única, personal e intransferible, y la construcción del diálogo en el grupo promovió la aceptación e incluso una convivencia positiva con el fenómeno.

## INTRODUCTION

The phenomenon of hearing voices can be defined as the experience of auditory hallucinations linked or not to visions, smells, touches or other sensations, such as hearing a person, music, noises, seeing something, the sensation of having something on the skin, when others cannot hear, see and/or feel, occurring in the absence of external sensory stimuli^(1,2)^. They can have numerous clinical meanings, and are not necessarily predictors of psychiatric diagnoses^(2)^. It often begins in children and adolescents^(3,4)^, and investigations with this group are necessary.

There is no epidemiological consensus on people who hear voices, with only estimates ranging from 5% to 15% of adults^(5-6)^ and 8% to 35% of children and adolescents^(3,5-7)^. Although being more common in children and adolescents, they tend to be transient in 95% of cases, with a reduction throughout development^(3,4)^. This aspect reiterates that hearing voices is not, in itself, a marker of psychiatric diagnosis, with only 25% of people who hear them meeting relevant criteria for this^(4)^. Due to this, children are referred to the Child and Adolescent Psychosocial Care Center (In Portuguese, *Centro de Atenção Psicossocial Infantojuvenil* - CAPSij), often without this being necessary. Characteristics and severity are variable, and it is crucial to establish a dialogue that favors the reception of both children, in relation to hearing the voice or voices, and family members, with children’s experience from the beginning^(3)^.

Historically, hearing voices was not always considered a bad thing. Civilizations attributed different meanings and senses to it, from demonic possession, holiness and witchcraft, until, in the 19^th^ century, with the rise of classical psychiatry, it began to be understood as a symptom of an illness^(6)^. The phenomenon of hearing voices, when captured by psychiatric knowledge, resulted in the pathologization of people who live this experience, regardless of age group^(7)^. This historical construction needs to be reinterpreted, as it does not validate individuals’ narratives, but rather minimizes them by labeling them. To achieve this, a long path needs to be traveled.

In addition to this aspect, children’s and adolescents’ mental health was also influenced by a psychiatric vision, with an ideal of protection that resulted in the construction of a care model based on institutionalization, segmentation, segregation, minimization of demands, and labeling, with the pathologization of childhood^(8,9)^. In this context, a child who hears voices tends to have their care demands suppressed in a context that makes them invisible, which can lead to suffering and care demands in adulthood. It is necessary to rethink this context, aiming to demonstrate that childhood will never be deprived of its own sensations and senses.

Thus, this study arises from the need to establish a dialogue with society regarding children’s experiences with hearing voices. Childhood is considered a social category of cultural production^(10,11)^ that must be visible. Recently, an integrative review identified scientific production regarding hearing voices in childhood, demonstrating a prevalence of quantitative studies, conducted in North America and Europe, focusing on epidemiological aspects, genetic and environmental factors^(12)^.

In this regard, building other understandings and approaches to care for the phenomenon of hearing voices indicates the need to know more about the experience from its perspective, particularly how it may be in nature, referring to how listeners determine the experience: whether voices, sounds, noises, visions, colors occur, where they come from (are they inside or outside the head), or whether they are somewhere else in the body; characteristics refer to the description made by listeners, whether gender, age, name, voice timbre and frequency are identified, whether there is more than one voice and whether they are friendly or aggressive; and the history of voices that is related to the beginning of the experience, whether a child can relate them to circumstances or events in life, how old they were when they started and whether there is any relationship with trauma^(13)^.

## OBJECTIVES

To understand narratives of children who hear voices, through their nature, characteristics and stories.

## METHODS

### Ethical aspects

This is a subproject of a study entitled “*Avaliação do Centro de Atenção Psicossocial Infantojuvenil do estado do Rio Grande do Sul (CAPSi-SUL)*”, which was approved by the Research Ethics Committee of the School of Nursing of Pelotas, funded by the Brazilian National Council for Scientific and Technological Development. The ethical precepts of Resolutions 466/2012 and 510/2016 of the Brazilian National Health Council were respected. To maintain anonymity, children chose names to identify them, such as colors, flowers and anime characters.

### Theoretical-methodological framework

The data were analyzed in light of Walter Benjamin’s concepts of memory and narrative^(14)^. For this author, narratives are a form of expression of subjectivity, because by promoting them, the meaning of one’s own existence is rescued. The author emphasizes that the narrative focus is the one that tells the story from one’s point of view. Memory is not seen by the author as a mere individualistic reaction of nostalgia, longing or empathy, but rather as remembrance and recollection that can be individual, but that helps the collective because it is a construction of the being.

### Study design

This is a narrative and qualitative study. To guide the writing of this study, the instrument COnsolidated criteria for REporting Qualitative research was used^(15)^.

### Study place, period and population

Initially, the study was scheduled to take place in person at a CAPij in the city of Pelotas, Rio Grande do Sul. However, due to the coronavirus disease (COVID-19) pandemic, it was necessary to adapt data collection to an online format. This collection took place through a virtual group called “Children United” (named after the wishes of children involved), led by the lead researcher. The group was created in July 2020 and was intended to welcome children (between 9 and 12 years old) who heard voices and their experiences, being the first Brazilian group of which we are aware. Data production was carried out using the participant observation technique, in all 96 meetings, between July 2020 and September 2021.

The recruitment period took place between May and July 2020. In total, six children were invited to participate in the study, with two recruited online and four referred by CAPSij. Two family members of children linked to CAPSij did not answer the researcher’s calls. In the end, four children were included in this study.

### Eligibility criteria

To select the interlocutors, school-age children (between 9 and 12 years old) who declared themselves to be voice hearers, had access to the internet, had authorization from their legal guardian to participate in the study of the “Children United” group, and had participated in the group for at least 15 meetings were included. It should be noted that, because the phenomenon of hearing voices is a multisensory experience, children who experienced voices, visions, smells, and/or other sensations remained in the study. No exclusion criteria were established.

### Data collection

To recruit participating children, the snowball sampling technique was used, where researchers created a group folder and shared it on their social networks. Family members who were interested in the group contacted the lead researcher via WhatsApp^®^. The researcher made a phone call, introduced the group and the study, invited them to participate and requested authorization. The family member was also asked if they knew any other children who could be included in the study. Dissemination was also carried out at CAPSij in the city of Pelotas, with professionals referring eligible children.

To formalize participation in the study, online acceptance was requested from the legal guardian through the Informed Consent Form, and from the child, through the Assent Form, both indexed on the Google Forms^®^ platform. Upon acceptance to participate, a WhatsApp^®^ group was created with the participation of children. The name “Children United” was chosen by the participating children, justified by its relationship with sharing and joint reflection of experiences, being an open group, with the possibility of including other children who were interested in participating. However, there was no inclusion of new participants in the study.

The “Children United” group took place twice a week, with all meetings held online, via video calls on the WhatsApp^®^ platform, lasting an average of 60 minutes, led by the main researcher (nurse and specialist in new approaches to child and adolescent mental health, PhD student in nursing, with extensive experience in conducting qualitative studies with children and at CAPSij). The group consisted only of the participating children and the researcher. No pilot test of the group was carried out, considering the researcher’s care experience.

Initially, a presentation of the group, the researcher and the members was made. Then, meetings were organized with the aim of mapping children’s experience of hearing voices. Data production occurred during the conduct of peer support groups, using the participant observation technique. In addition, individual narrative interviews were conducted online and recorded with a media recorder, with a date and time previously agreed with children. The interviews were transcribed by the researcher.

To guide the meeting and the narrative about hearing voices, the researcher used open-ended questions such as: tell me about your experience with the voices, visions and/or sensations, when it started, what you were doing. Data production was completed in September 2021, due to the robustness of the material produced during the 96 meetings, supplemented by the interview, with consensus among researchers. After data production was completed, the group remained active in order to maintain support activities for children.

It is worth reiterating that, during the meetings, children often moved away from the main topic and addressed other care needs, with the researcher allowing this so that the bond between them could be maintained and the focus (listening to voices) could be addressed in its entirety at later times. Furthermore, notes were made in a field diary during and after the meetings, which contributed to the systematic composition of children’s narratives about the experience of hearing voices.

As the meetings took place, each month, the researchers discussed the progress of the investigation, aiming to improve the group and data production. No prior relationship was established with children, only a bond was established during the meetings. It is also worth noting that the WhatsApp^®^ group was a means of communication only about the meetings.

### Data analysis

The meetings were recorded with an audio recorder, with subsequent transcription of the speeches of children involved in the interviews through narratives that responded to the research objective. The transcriptions were carried out by the main researcher, with subsequent validation by another researcher. In data analysis, the concepts of memory and narrative were used^(14)^, in addition to the proposed data segmentation^(16)^. To this end, field diary notes and field notes were read exhaustively, segmenting the materials into concrete experiences and identifying references that expressed beliefs, value judgments, and life wisdom. Empirical materials were then individualized with concrete experiences and references to compose narratives about hearing voices in childhood based on the nature of experiences, the characteristics of voices, and the history of voices. To assist this process, the N vivo 10 Windows^®^ software was used in all the aforementioned stages, enabling data organization and categorization.

Analysis was conducted independently by two researchers, with subsequent discussion with the other researchers, reaching a consensus. In the end, three categories emerged, with excerpts from the interviews indicated in the results: 1) Children’s multisensory experience: nature of experiences; 2) Hearing voices: an unusual beginning; and 3) Voice characteristics: who are they?

## RESULTS

### Participant characterization

Four children participated in this study: Naruto was 9 years old and was being followed up at an occupational therapy service recommended by social media; Blue was 12 years old and was not being followed up at a healthcare service, except for other situations, referred to the group by social media and living in the state of Minas Gerais; Sunflower was 12 years old, followed up at CAPSij, having been referred by the service; finally, Jedai was 9 years old, followed up at CAPSij, also referred by the service to participate in the group. The categories and their narratives will be presented below.

#### 1) Children’s multisensory experience: nature of experiences

The interlocutors presented diverse experiences regarding the nature of voices. All children heard voices, but only Jedai did not experience visions, and the others reported episodes of visions throughout the day. Blue shared that he could not identify where the voices came from, whether they were internal or external, appearing in a nonspecific manner, but he could clearly identify where his visions came from. Naruto identified the nature of voices as internal, with characteristics of commands about what he should do. Sunflower reported that the voices and visions were simultaneous, identifying where they came from, when they appeared, the frequency and their relationship with the experience (Chart 1).

**Chart 1:** Onset, nature and characteristics of experiences

Participating child	Categories		
	1) Children’s multisensory experience: nature of experiences	2) Hearing voices: an unusual beginning	3) Voice characteristics: who are they?
**Blue**	“*The images were specific, but I don’t know the specific direction of the voices. I can identify the visions, which were of a woman and a man. They didn’t always appear together. Often it was just the woman, and other times it was just the men. She was wearing a white dress, and he was wearing a plaid shirt and had a beard*”.	“*I don’t remember, I don’t remember the beginning, they came much earlier, after I started Reiki* [to deal with the voices], *and learned lessons to take care of myself, the power of the mind.* [...] *it was really bad, it scared me, the visions appeared, I was scared, they appeared out of nowhere*”.	“*A woman and a man would appear in the kitchen, in the bathroom, and before they could say anything to me, I would run away*. [...] *I can remember that the person sitting in the chair was a child, but the others were older adults. The lumberjack was an older adult, the woman in white, I don’t know how old she was, they would appear quite a lot. Over time, they have been decreasing*”.
**Naruto**	“*They* [voices] *come from outside my body. They appear late at night, from time to time, when I’m playing, they tell me to see how the game is going, to be quiet to play*”.	“*I can’t remember, but it was one afternoon. I was on my cell phone; I was watching a video.* [...] *if I can relate it to anything, it’s that I have a dog that died. They poisoned him in the yard, about six or seven years ago, I was at school and I arrived. I always remember him and sometimes I miss him a lot”.*	“*I have two voices, Joãozinho and Juviscláudio, both of them appeared at different times. Joãozinho is a boy, Juviscláudio is the older one, they are good, but they tell me to do things. Now, Joãozinho is the same age as Juviscláudio, they are both 26 years old, they both speak very calmly, with a calm tone of voice, they talk to me every day*”.
**Sunflower**	“*The voices came into my head, sometimes they were outside, while I was using my cell phone. They also appeared during recess, during school hours, I spoke very loudly to my classmates so they wouldn’t know I was listening*”.	“*They started at the beginning of November. It started when I was 9 years old, so I was at school* [...] *I heard the voices at school. During recess, there was a male voice and a female voice. They were inside my head, and what preceded the start at that moment was a strong sensation, as if something was going to happen, I was scared and told my friend*”.	“*They didn’t have names, they were children, they were 13 years old, girls, they spoke softly, they showed up for a few days, but then I didn’t have them anymore*”.
**Jedi**	“*I don’t know where the voices were coming from, it seemed to come from the street, from outside my head, it sounded like a monster*”.	“*I can’t remember when it started, but I remember that my mother took me to CAPSi after it started, because I was also very agitated*”.	“*There was a hand on the window and I thought it was a monster and I was very scared, we ran to tell my father, who didn’t believe us, it was night*. [...] *another time, my mother was going to take me and my brother to school, we were walking and I dropped my jaw, when I heard a voice that sounded like a Lego voice. I looked around and behind me, and there was no one there*”.

#### 2) Hearing voices: an unusual beginning

As for the beginning of the experience with the voices, the reports presented covered the month of onset, the time of day and activities carried out, such as being at school or on the way to school. They narrated bodily sensation, described by a child as a strong feeling that something was about to happen, as well as the emotion experienced described as fear. Another situation was the event about the death of Naruto’s pet dog (Chart 1).

#### 3) Voice characteristics: who are they?

Children described in their narratives the names of some of the voices, their exact age or age range, gender, tone, frequencies, and what they said to them. Specifically, only Naruto heard, had visions, and felt the presence of the voices daily, describing his relationship with them as friendly. For Blue and Sunflower, the voices appeared every few days. Blue narrated that the voices became less frequent over time, and for Sunflower, the voices disappeared over the course of the meetings (Chart 1).

## DISCUSSION

It was noted in this study that the nature of voices varied between internal and external. External voices are different from internal voices because they are heard from outside any physical organ, from different directions, allowing listeners to discern whether the voice is from an adult, an older adult or a child. This differentiation is seen in the intensity of the relationship established when the voice is internal, while in external voices there is a posture in the direction of the voice that is being heard^(17)^.

The experience of hearing voices is highly personal^(3,9,18)^, and only individuals can establish its nature and judge its meaning, requiring the establishment of some understandings, such as the sensory nature, based on the assumption that they are not thoughts, but true perceptions, similar to hearing the interviewer’s voice^(2)^.

At the beginning of each listener’s experience, the voices are only heard and not listened to, because listening demands voluntary attention to what is being said, which is not the case for most listeners, especially at the beginning of the hearing process. Listeners tend not to seek explanations about what is happening, because the experience, for some children, is terrifying^(17)^. Another aspect concerns the diversity of the phenomenon, since in this study, all children hear voices, but they also present visions and sensations that must be considered in the investigation of their nature. The importance of dialogue in the group is understood, but especially from the beginning of the phenomenon so that the experience is not traumatic, since the continuity of the phenomenon may be a reality and it will be necessary for the relationship to be welcomed in order to establish an adequate coexistence.

The phenomenon of hearing voices can often be associated with the idea of an “imaginary friend”, which is common in child and adolescent development. In this case, children construct complex narratives creatively and sociably. In a cross-sectional European study, it was observed that, of 1,472 adults, 41% had an imaginary friend in childhood; of these, 69% were associated with hearing voices and 61% with seeing and feeling them. However, no association was found between this experience and hallucinations in adulthood^(18)^, which demonstrates that hearing voices, in itself, is not an indication of future problems.

In this study, children narrated the voice characteristics in depth, and some aspects were observed, such as noticing whether there was a lack of control over the voices, confirming that there was no obvious external auditory stimulus, observing whether the noises or voices consisted of a name being called, or whether the voices were addressed to children. There is a portion of voice listeners who are able to live positively with these experiences^(18)^, but it is a complex process for children to develop. Fear was the emotion most experienced in narratives of the phenomenon, which supports current literature^(12,16,18-20)^. These reactions may be associated with voice characteristics, such as the meaning attributed to the voices (intrusive, controlling and threatening)^(20,21)^ and the number of voices. The occurrence was also seen in a study with 52 participants, with 20% reporting hearing one voice, 53% between two and four voices, 10% between five and 10 voices, 8% between 11 and 20 voices, and 9% more than 20 voices, with the number being associated with negative repercussions, mainly with command of actions^(19)^.

When faced with the history of voices, their understanding emerges from the context of each interlocutor’s life. The countless situations and crossings that are related to the beginning, continuity or cessation of the experience realized that, when narrating their stories, an individual process began, allowing interlocutors to look at themselves and their memories, in addition to recognizing the different moments in which the voices appeared and their associations with daily life. These aspects contributed to the possibility of rediscovering their stories.

Some events, such as the poisoning of Naruto’s dog and being at school for Sunflower, have been identified as situations that triggered voices and visions. However, there is no way to confirm the existence of a cause for the appearance of voices in the lives of all people who hear them or even that it could be the expression and production of subjectivity, but it is likely to happen at a very young age.

As mentioned above in the introduction to this manuscript, voices in children tend to be transient, as seen in Sunflower’s account. It is estimated that approximately three-quarters of young people stop hearing voices within the first five years of their onset^(7)^. A case-control study with 145 cases (voice hearers) and 148 controls indicated that hearing voices remained in 18.2% of cases at 6 years of age, decreasing to 6.2% at 11 years of age^(19)^, and decreasing with age. The literature indicates that voices may disappear for a period and then return, especially in cases of adversity in childhood^(12)^, but this should not be seen as a problem, as long as a child learns to create a peaceful coexistence with the hearing experience.

However, this process can be hindered by social representation and historical influence of conventional psychiatric labels regarding hearing voices, which are still permeated by stigmas^(21,22)^ and the pathologization of childhood. Qualitative research conducted with adolescents in the United States demonstrated that they are aware of stigmas associated with hearing voices, which is a source of suffering. Stigma is, in its prevalence, associated with exclusion for being “different,” and this labeling tends to make children view the voice negatively, with diverse emotional reactions to the experience, and not seeking healthcare services for support, for fear of professional stigmatization^(7)^.

Fear of stigma among adolescents by healthcare professionals should be considered, since many professionals still work in a biomedical and reductionist logic. A study conducted with physicians^(23)^ demonstrated that they represent the phenomenon of hearing voices in childhood as a psychiatric diagnosis, with stigma and reluctance in providing care. It is worth considering that this view was seen in primary and secondary care physicians, places that should be the organizing center of care for this adolescent^(9)^, mainly in health promotion. The same is seen in a meta-synthesis conducted with studies on nurses’ perceptions, who also demonstrated fear of aggression and, consequently, distancing themselves from the care provided^(22)^. It is known that this perspective can be initiated from professionals’ graduation, being of vital importance the inclusion of the topic in their training^(24)^.

The aforementioned views have repercussions on care. A cross-sectional study^(3)^ conducted with 95 children who heard voices showed that: 52.6% had more than one psychiatric diagnosis and that 38.9% had a psychiatric diagnosis, demonstrating the phenomenon of pathologization of childhood; 11.6% used antipsychotic medications, which may demonstrate the phenomenon of medicalization of childhood; and only 19% were referred to a psychotherapy group, which, in a line of care, would be the most empowering approach, as carried out by the “Children United” group.

Another point is that stigma also follows families, influenced by the community’s view of the phenomenon. A study conducted with family members noted that they interpreted their children’s experience as “dangerous” and “scary”, associating it with psychiatric diagnoses and acting with child overprotection^(6)^, a fact that is detrimental to their full development. It is worth highlighting that this study also involved adolescents, who reported not feeling comfortable talking about the experience due to their families’ views^(6,25)^. In this process, family groups are necessary in the Psychosocial Care Network (In Portuguese, *Rede de Atenção Psicossocial* - RAPS) facilities to enable family support and, consequently, promote children’s well-being.

When a child tells someone that they are hearing voices, they may be seeking a source of support and comfort to help them live with the experience^(26)^, making it necessary to overcome the stigma and build a space that is welcoming to their needs^(27)^. Although the “Children United” group is not the focus of this study, it is worth highlighting its potential as a space for care. The interlocutors reflected on many issues regarding their experiences, and the group’s support offered strategies on how to deal with them. Thus, it was possible for children, in contact with their subjective experiences, to experience insights to deal with the emotions present and, consequently, with their own lives. As mentioned previously, children tend to omit hearing voices due to their representation in the social context; for this, it takes time for children to create a bond and perceive the security of narrating the phenomenon and becoming a leading figure of this story, and the group demonstrated potential for this. For future studies, it is worth reflecting on its applicability in RAPS equipment.

Several authors^(7,13,19,21)^ reported that when children assume that they are voice hearers, they may consider it just a different way of experiencing the world around them; it is like a human variation, because, just as there are right-handed and left-handed people, there are people who have experience with voices, visions, smells and other sensations^(13,28)^. It is important for children to be able to discover what actually helps in this process of establishing contact with the experience^(6,12,28)^, as it takes time for people to accept that they are voice listeners^(13)^. It is necessary that individual narratives of nature, characteristics and history are seen and reproduced in clinical practice and in other studies, moving towards the redefinition of the phenomenon.

### Study limitations

This study has the following limitations: the number of participants, although these are rich narratives from children who participated in a considerable number of meetings; the online data collection approach, considering that many children may feel shy about expressing their perceptions, especially under family supervision, and the reach of children being limited to those who had cell phones and internet; and the fact that data production was centralized in two Brazilian states (Rio Grande do Sul and Minas Gerais). For future studies, it is recommended to expand the collection of experiences, considering that culture is a determinant of health.

### Contributions to health, nursing or public policy

Narratives in this study are of vital importance for advancing literature on voice-hearing in children, demonstrating the nature, characteristics and stories that can guide healthcare professionals’ work in the construction of unique therapeutic projects. Furthermore, this study goes against conventional and biomedical frameworks, moving from the logic of pathologizing childhood to the moment of giving new meaning the hearing of voices, giving meaning to the experiences and space for narratives of children.

## FINAL CONSIDERATIONS

This study sought to understand the experiences of children who hear voices through their narratives, and from these, it was possible to observe that children experience voices, visions, smells and sensations in a unique, personal and non-transferable manner. Given the nature of experiences, it was noted that all children had voices and not all had visions or other sensations. Children indicated when they began hearing voices, especially in relation to the month, time of day and activities carried out at the time. When indicating the voice characteristics, it was possible to observe the description of gender, age range, tone of voice, frequency and content of voices/visions. In this study, the construction of dialogue about the experiences of hearing voices promoted acceptance and even a positive coexistence with the phenomenon. The group’s potential as a space for dialogue with children is believed, since promoting children’s leading role means cultivating new ways of accepting the experience of hearing voices.

## Data Availability

The research data are available within the article.
