# Supplementary material for: Narratives of children who hear voices: nature, characteristics and stories
Source: Rev Bras Enferm. 2025 Nov 21;78(Suppl 2):e20240314. doi: 10.1590/0034-7167-2024-0314 (PMC12659911; doi:10.1590/0034-7167-2024-0314)
Supplement: Translation

# Translation: Narrativas de crianças que ouvem vozes: natureza, característica e histórias

**DOI:** 10.1590/0034-7167-2024-0314pt

**Authors:** Clarissa de Souza Cardoso, Valéria Cristina Christello Coimbra, Luciane Prado Kantorski, Milena Hohmann Antonacci, Danton Matheus de Souza, Liamara Denise Ubessi

**Affiliations:** I Universidade Federal do Pampa. Uruguaiana, Rio Grande do Sul, Brasil; II Universidade Federal de Pelotas. Pelotas, Rio Grande do Sul, Brasil; III Universidade de São Paulo. São Paulo, São Paulo, Brasil

**Keywords:** Saúde Mental, Saúde da Criança, Apoio Social Online, Enfermagem Pediátrica, Promoção da Saúde.

## Abstract

**Objetivos:**

compreender as narrativas de crianças que escutam vozes, por meio da sua natureza, característica e histórias.

**Métodos:**

estudo narrativo com abordagem qualitativa. Utilizaram-se os conceitos benjaminianos de narrativa e memória para analisar a contribuição de quatro crianças autodeclaradas ouvintes de vozes.

**Resultados:**

das narrativas, emergiram três categorias: 1) A experiência multissensorial das crianças: natureza das experiências (todas possuíam vozes, nem todas tinham visões ou outras sensações); 2) Ouvir vozes: um começo inusitado (as narrativas versaram sobre mês de início, turno do dia e atividades realizadas no momento); e 3) Características das vozes: quem são elas? (foram descritos o gênero, faixa etária, tom de voz, frequência e conteúdo das vozes/visões).

**Considerações Finais:**

observou-se, a partir das narrativas, que as crianças experimentam as vozes, visões e sensações de forma única, pessoal e intransferível, e a construção do diálogo no grupo promoveu acolhimento e até mesmo uma convivência positiva com o fenômeno.

## INTRODUÇÃO

O fenômeno de ouvir vozes pode ser definido como a vivência de alucinações auditivas ligada ou não a visões, cheiros, toques ou outras sensações, como ouvir uma pessoa, músicas, ruídos, ver algo, sensação de ter algo sobre a pele, quando outros não conseguem ouvir, ver e/ou sentir, ocorrendo na ausência de estímulos externos sensoriais^(1,2)^. Podem ter inúmeros significados clínicos, e não necessariamente são preditores de diagnósticos psiquiátricos^(2)^. É frequente que o seu início seja na faixa infantojuvenil^(3,4)^, sendo necessárias investigações com esse público.

Não há um consenso epidemiológico sobre as pessoas que ouvem vozes com apenas estimativas, que variam entre 5% e 15% dos adultos^(5,6)^ e 8% e 35% de crianças e adolescentes^(3,5-7)^. Apesar de mais frequentes no público infantojuvenil, elas tendem a ser transitórias em 95% dos casos, com redução ao longo do desenvolvimento^(3,4)^. Esse aspecto reitera que ouvir vozes não é, por si só, um marcador de diagnóstico psiquiátrico, sendo que apenas 25% das pessoas que as ouvem atendem a critérios relevantes para tal^(4)^. Devido a isso, crianças são encaminhadas ao Centro de Atenção Psicossocial Infantojuvenil (CAPSij), muitas vezes sem haver esta necessidade. As características e gravidade são variáveis, sendo crucial estabelecer um diálogo que favoreça o acolhimento tanto da criança, em relação à escuta da voz ou das vozes, quanto dos familiares, com a experiência da criança desde o início^(3)^.

Historicamente, nem sempre ouvir vozes foi considerado algo ruim. As civilizações atribuíam significados e sentidos diferentes, desde possessão demoníaca, santidade e bruxaria, até que, no século XIX, com a ascensão da psiquiatria clássica, passou a ser compreendida como sintoma de uma doença^(6)^. O fenômeno da escuta das vozes, ao ser capturado pelo saber psiquiátrico, resultou na patologização das pessoas que vivem essa experiência, independentemente da faixa etária^(7)^. Essa construção histórica precisa ser ressignificada, pois não valida as narrativas dos indivíduos, mas as minimizam, colocando-as em rótulos. Para tal, um longo caminho precisa ser percorrido.

Somado a esse aspecto, a saúde mental infantojuvenil também foi influenciada por uma visão psiquiátrica, com um ideário de proteção que redundou na construção de um modelo de assistência pautado na institucionalização, segmentação, segregação, minimização das demandas e rotulação, com a patologização da infância^(8,9)^. Nesse caminhar, uma criança que ouve vozes tende a ter suas demandas de cuidado oprimidas em um contexto que as invisibiliza, o que pode repercutir em sofrimento e demandas de cuidado na fase adulta. É necessário repensar esse contexto, visando demonstrar que a infância jamais estará privada de suas sensações e sentidos próprios.

Assim, este estudo nasce da necessidade de estabelecer um diálogo com a sociedade em relação às experiências infantis com a escuta de vozes. A infância é considerada uma categoria social de produção de cultura^(10,11)^ que deve ter visibilidade. Recentemente, revisão integrativa identificou a produção científica a respeito da escuta das vozes na infância, demonstrando uma prevalência de estudos quantitativos, conduzidos na América do Norte e Europa, com enfoque em aspectos epidemiológicos, fatores genéticos e ambientais^(12)^.

Nesse sentido, construir outros entendimentos e abordagens de cuidado diante do fenômeno de ouvir vozes indica a necessidade de conhecer mais a respeito da experiência em sua perspectiva, particularmente, como ela pode ser na natureza, referindo-se à como o ouvinte determina a experiência: se ocorrem vozes, sons, ruídos, visões, cores, de onde elas vêm (estão do lado de dentro ou de fora da cabeça), ou ainda se estão em outro lugar do corpo; as características referem-se à descrição feita pelo ouvinte, se identifica o gênero, idade, nome, timbre de voz e a frequência, se existe mais de uma voz e se são amigáveis ou agressivas; e a história das vozes que está relacionada ao início da experiência, se a criança consegue relacionar a circunstâncias ou acontecimentos na vida, quantos anos ela tinha quando iniciaram e se há alguma relação com traumas^(13)^.

## OBJETIVOS

Compreender as narrativas de crianças que escutam vozes, por meio da sua natureza, característica e histórias.

## MÉTODOS

### Aspectos éticos

Trata-se de subprojeto do estudo “Avaliação dos Centros de Atenção Psicossocial Infantojuvenil do estado do Rio Grande do Sul (CAPSi-SUL)”, que foi aprovado pelo Comitê de Ética em Pesquisa da Faculdade de Enfermagem de Pelotas, financiado pelo Conselho Nacional de Desenvolvimento Científico e Tecnológico. Respeitaram-se os preceitos éticos das Resoluções N° 466/2012 e n° 510/2016 do Conselho Nacional de Saúde. Para manter o anonimato, as crianças escolheram nomes para identificá-las, como cores, flores e personagens de animes.

### Referencial teórico-metodológico

Os dados foram analisados à luz dos conceitos de memória e narrativa de Walter Benjamin^(14)^. Para este autor, as narrativas são uma forma de expressão da subjetividade, pois, ao promovê-las, resgata-se o sentido da própria existência. O autor destaca que o foco narrativo é aquele que narra a história sob o seu ponto de vista. A memória não é vista pelo autor como mera reação individualista da nostalgia, da saudade ou de empatia, mas sim como rememoração e lembrança que pode ser individual, mas que auxilia no coletivo por ser uma construção do ser.

### Delineamento do estudo

Estudo qualitativo, do tipo narrativo. Para guiar a redação deste estudo, utilizou-se o instrumento *Consolidated Criteria for Reporting Qualitative Research* ^(15)^

### Local, período e população de estudo

Inicialmente, o estudo estava programado para ocorrer de forma presencial em um CAPSi no município de Pelotas, Rio Grande do Sul. Contudo, devido à pandemia da doença do coronavírus (COVID-19), foi necessário adaptar a coleta de dados para a modalidade *online*. Essa coleta ocorreu por meio de um grupo em ambiente virtual intitulado “Crianças Unidas” (denominação a partir do desejo das crianças envolvidas), guiado pela pesquisadora principal. O grupo foi criado em julho de 2020, e foi destinado a acolher crianças (entre 9 e 12 anos incompletos) que ouviam vozes e suas experiências, sendo o primeiro grupo brasileiro do qual se tem conhecimento. A produção de dados ocorreu pela técnica de observação participante, em todos os 96 encontros, entre julho de 2020 e setembro de 2021.

O período de recrutamento ocorreu entre os meses de maio e julho de 2020. No total, seis crianças foram convidadas para participar do estudo, com duas recrutadas pelo meio *online* e quatro encaminhadas pelo CAPSi. Dois familiares de crianças vinculadas ao CAPSi não atenderam as ligações da pesquisadora. Ao fim, quatro crianças foram incluídas neste estudo.

### Critérios de elegibilidade

Para seleção dos interlocutores, foram incluídas crianças em idade escolar (entre 9 e 12 anos incompletos), que se autodeclararam ouvintes de vozes, com acesso à internet, autorização do responsável legal para participar do estudo do grupo “Crianças Unidas” e participação no grupo por, no mínimo, 15 encontros. Ressalta-se que, pelo fenômeno de ouvir vozes ser uma experiência multissensorial, as crianças que vivenciavam vozes, visões, cheiros e/ou outras sensações se mantiveram no estudo. Não foram estabelecidos critérios de exclusão.

### Coleta de dados

Para o recrutamento das crianças participantes, utilizou-se a técnica de amostragem bola de neve, onde os pesquisadores criaram um *folder* do grupo e divulgaram em suas redes sociais. Os familiares que se interessavam pelo grupo entravam em contato com a pesquisadora principal pelo *WhatsApp* ^®^. A pesquisadora realizava uma ligação telefônica, apresentando o grupo e o estudo, com o convite à participação e solicitação da autorização. Ainda, o familiar era questionado se conhecia outra criança que poderia ser incluída no estudo. A divulgação também foi realizada nos CAPSi do município de Pelotas, com o encaminhamento de crianças elegíveis pelos profissionais.

Para a oficialização da participação no estudo, foi solicitado um aceite *online* da responsável legal por meio do Termo de Consentimento Livre e Esclarecido, e da criança, por meio do Termo de Assentimento, ambos indexados na plataforma *Google Forms* ^®^. Depois do aceite de participação, criou-se o grupo no *WhatsApp* ^®^ com a inclusão das crianças participantes. O nome “Crianças Unidas” foi escolhido pelas crianças participantes, justificado pela sua relação com o compartilhamento e reflexão conjunta das experiências, sendo um grupo aberto, com a possibilidade de inclusão de outras crianças que tivessem interesse em participar. Contudo, não houve a inclusão de novos participantes no estudo.

O grupo “Crianças Unidas” ocorria duas vezes por semana, com todos os encontros realizados de forma *online*, por chamadas de vídeo na plataforma *WhasApp* ^®^, com média de duração de 60 minutos, guiados pela pesquisadora principal (enfermeira e especialista em novas abordagens em saúde mental infantojuvenil, doutoranda em enfermagem, com ampla experiência na condução de estudos qualitativos com crianças e no CAPSi). O grupo era mantido apenas com as crianças participantes e a pesquisadora. Não foi realizado teste piloto do grupo, considerando a experiência assistencial da pesquisadora.

Inicialmente, foi realizada uma apresentação do grupo, da pesquisadora e dos membros. Em seguida, foram organizados encontros com o objetivo de mapear a experiência das crianças com a escuta das vozes. A produção de dados ocorreu durante a condução dos grupos de suporte por pares, utilizando a técnica de observação participante. Além disso, foram realizadas entrevistas narrativas individuais *online* e gravadas com um gravador de mídia, com data e horário combinados previamente com as crianças. As entrevistas foram transcritas pela pesquisadora.

Para guiar o encontro e a narrativa sobre a escuta das vozes, a pesquisadora fazia uso de perguntas abertas como: conte-me sobre sua experiência com as vozes, visões e/ou sensações, quando começou, o que você estava fazendo. A produção dos dados foi finalizada em setembro de 2021, devido à robustez do material produzido durante os 96 encontros, complementados pela entrevista, havendo consenso entre as pesquisadoras. Após o término da produção dos dados, o grupo permaneceu ativo com o intuito de manter as atividades de suporte às crianças.

Reitera-se que, nos encontros, as crianças, frequentemente, afastaram-se do tema principal e abordaram outras necessidades de cuidado, sendo permitido pela pesquisadora, para que o vínculo entre eles se mantivesse e o enfoque (escuta das vozes) fosse abordado em sua integralidade em momentos posteriores. Ainda, foram realizadas anotações em um diário de campo durante e após a realização dos encontros, que contribuíram para a composição sistemática das narrativas das crianças sobre a experiência com a escuta de vozes.

Conforme os encontros ocorriam, mensalmente, os pesquisadores discutiam o caminhar da investigação, visando ao aperfeiçoamento do grupo e da produção de dados. Não foi estabelecido um relacionamento prévio com as crianças, apenas o vínculo ao longo dos encontros. Ainda, é digno de nota que o grupo de *WhatsApp* ^®^ foi um veículo de comunicação apenas sobre os encontros.

### Análise dos dados

Os encontros foram gravados com gravador de áudio, com posterior transcrição dos discursos das crianças interlocutoras por meio das narrativas que respondessem ao objetivo da pesquisa. As transcrições foram realizadas pela pesquisadora principal, com validação posterior por outro pesquisador. Na análise dos dados, utilizaram-se os conceitos de memória e narrativa^(14)^, somada à proposta de segmentação dos dados^(16)^. Para tal, realizou-se a leitura exaustiva das anotações em diário de campo e das notas de campo, com segmentação dos materiais em experiências concretas e identificação das referências que expressassem as crenças, juízos de valores e sabedoria de vida. Em seguida, os materiais empíricos foram individualizados com as experiências concretas e referências, para compor as narrativas sobre ouvir vozes na infância com base na natureza das experiências, nas características das vozes e na história das vozes. De forma a auxiliar esse processo, foi utilizado o *software* N vivo 10 *Windows^®^* em todas as etapas supracitadas, possibilitando uma organização dos dados e sua categorização.

A análise foi conduzida por dois pesquisadores, de forma independente, com posterior discussão com os demais pesquisadores, chegando a um consenso. Ao fim, emergiram três categorias, com a indicação nos resultados de trechos das entrevistas: 1) A experiência multissensorial das crianças: natureza das experiências; 2) Ouvir vozes: um começo inusitado; e 3) Características das vozes: quem são elas?

## RESULTADOS

### Caracterização dos participantes

Quatro crianças participaram deste estudo: Naruto tinha 9 anos e realizava acompanhamento em um serviço de terapia ocupacional indicado pelas redes sociais; Azul tinha 12 anos e não realizava acompanhamento em serviço de saúde, exceto para outras situações, encaminhado para o grupo pelas redes sociais e residia no estado de Minas Gerais; Girassol tinha 12 anos, acompanhada no CAPSi, tendo sido indicada pelo serviço; por fim, Jedai tinha 9 anos, acompanhado no CAPSi, também indicado pelo serviço para a participação no grupo. A seguir, serão apresentadas as categorias e suas narrativas.

#### 1) Experiência multissensorial das crianças: natureza das experiências

Os interlocutores apresentam experiências diversificadas diante da natureza das vozes. Todas as crianças ouviam vozes, mas apenas Jedai não vivenciava visões e as demais referiram episódios com visões ao longo do dia. Azul compartilhou que não identificava de onde vinham as vozes, se eram internas ou externas, surgindo de forma inespecífica, mas identificava de onde vinham suas visões de forma nítida. Naruto identificou a natureza das vozes como internas, com características de comandos sobre o que deveria fazer. Girassol relatou que as vozes e visões eram simultâneas, identificando de onde vinham, qual o momento que apareciam, a frequência e sua relação com a experiência (Quadro 1).

**Quadro 1:** Início, natureza e características das experiências

Criança participante	Categorias		
	1) Experiência multissensorial das crianças: natureza das experiências	2) Ouvir vozes: um começo inusitado	3) Características das vozes: quem são elas?
Azul	“*As imagens eram específicas, mas as vozes não sei direito a direção específica. Consigo identificar as visões, que eram de uma mulher e um homem, nem sempre apareciam juntos, muitas vezes era só a mulher, em outras, só os homens. Ela estava com um vestido branco, e ele, de camisa xadrez e barba*”.	“*Não lembro, tipo do início não lembro, vinham muito antes, depois que entrei no Reiki* [para lidar com as vozes], *e aprendi lições para se cuidar, poder da mente*. [...] *foi bem ruim, me assustou, as visões apareceram fiquei com medo, apareceram do nada*”.	“*Uma mulher e um homem apareciam na cozinha, no banheiro, antes que eles pudessem me dizer alguma coisa, eu saia correndo*. [...] *eu consigo lembrar que aquela* [voz] *da pessoa sentada na cadeira era criança, mas as outras eram idosos. O lenhador era idoso, a mulher de branco, não sei quantos anos tinha, elas apareciam bastante. De um tempo pra cá, foram diminuindo*”.
**Naruto**	“*Eles* [vozes] *vêm de fora do meu corpo. Eles aparecem tarde da noite, de vez em quando, quando estou jogando, eles falam para ver como que está o jogo, para ficar quieto para jogar*”.	“*Não consigo lembrar, mas foi em uma tarde. Eu estava mexendo no celular, eu estava olhando um vídeo*. [...] *se eu relaciono com alguma coisa, é que eu tenho um cachorro que morreu. Envenenaram ele no pátio, faz uns seis ou sete anos isso, eu estava na escolinha e eu cheguei. Eu lembro sempre dele e às vezes tenho muita saudade*”.	“*Eu tenho duas vozes, o Joãozinho e o Juviscláudio, os dois apareceram em diferentes momentos. O Joãozinho é um menino, o Juviscláudio é o mais velho, eles são bonzinhos, mas eles me falam pra fazer coisas. Agora, o Joãozinho está com a mesma idade do Juviscláudio, os dois estão com 26 anos, os dois falam bem calmo, com tom de voz tranquilo, eles falam comigo todos os dias*”.
**Girassol**	“*As vozes vinham na minha cabeça, às vezes estavam fora, nos momentos enquanto mexia no celular. Também apareciam no recreio, na hora da escola, eu falava bem alto com as minhas colegas para que elas não soubessem que eu estava ouvindo*”.	“*Iniciaram no começo de novembro. Começou quando eu tinha 9 anos, então eu estava na escola* [...] *eu ouvia as vozes no colégio. Na hora do recreio, tinha uma voz masculina e uma feminina. Elas estavam dentro da cabeça, que o que antecedeu o início naquele momento foi uma sensação forte, como se algo fosse acontecer, fiquei assustada e contei para o meu amigo*”.	“*Não tinham nome, eram crianças, elas tinham 13 anos, meninas, falavam baixo, apareciam em alguns dias, mas depois eu não tive mais*”.
**Jedai**	“*Não sei de onde vinham as vozes, parecia vir da rua, de fora da minha cabeça, parecia um monstro*”.	“*Eu não consigo lembrar quando foi que começou, mas eu lembro que a mãe me levou no CAPSi depois que começou, porque eu também tava muito agitado*”.	“*Tinha uma mão na janela e eu pensava que se tratava de um monstro e senti muito medo, fomos correndo contar para o meu pai, que não acreditou, era noite*. [...] *outra vez, a mãe estava indo me levar eu e o mano na escola, a gente estava indo a pé e cai de queixo, quando ouvi uma voz que parecia uma voz do lego. Eu olhei para os lados e para trás, e não tinha ninguém*”.

#### 2) Ouvir vozes: um começo inusitado

A respeito do início da experiência com as vozes, os relatos apresentados versaram sobre o mês de início, o turno do dia e as atividades realizadas, como estar na escola ou a caminho dela. Narraram sobre a sensação corpórea, qualificada pela criança como uma sensação forte de que algo estava prestes a acontecer, assim como a emoção experimentada descrita pelo medo. Outra situação foi o evento sobre a morte do cachorro de estimação do Naruto (Quadro 1).

#### 3) Características das vozes: quem são elas?

As crianças descreveram em suas narrativas o nome que algumas vozes possuíam, a idade precisa ou faixa etária, o gênero, o tom, as frequências e o que lhes diziam. Especificamente, apenas Naruto ouvia, tinha visões e sentia a presença das vozes diariamente, descrevendo a sua relação com elas como amigável. Já para Azul e Girassol, as vozes apareciam alguns dias. Azul narrou que as vozes foram reduzindo com o tempo, e para Girassol, as vozes sumiram no decorrer dos encontros (Quadro 1).

## DISCUSSÃO

Percebeu-se, neste estudo, que a natureza das vozes variou entre interna e externa. As vozes externas são diferentes das internas, por serem ouvidas de fora de qualquer órgão físico, a partir de diversas direções, permitindo que o ouvinte possa discernir se a voz é de um adulto, idoso ou criança. Essa diferenciação é vista na intensidade da relação estabelecida quando a voz é interna, enquanto nas vozes externas há uma postura na direção da voz que está ouvindo^(17)^.

A experiência com a audição de vozes é altamente pessoal^(3,9,18)^, e somente o indivíduo poderá estabelecer sua natureza e julgar seu significado, necessitando do estabelecimento de alguns entendimentos, como a natureza sensorial, partindo do pressuposto de que não são pensamentos, mas percepções verdadeiras, semelhantes a ouvir a voz do entrevistador^(2)^.

No início da experiência de cada ouvidor, as vozes são apenas ouvidas e não são escutadas, pois a escuta demanda atenção voluntária no que está sendo dito, o que não acontece com a maioria dos ouvidores, especialmente no início do processo de audição. O ouvidor tende a não procurar explicações sobre o que está acontecendo, pois a experiência, para uma parte das crianças, é aterrorizante^(17)^. Outro aspecto diz respeito à diversidade do fenômeno, pois, neste estudo, todas as crianças ouvem vozes, mas também apresentam visões e sensações que devem ser consideradas na investigação da natureza. Entende-se a importância do diálogo no grupo, mas sobretudo desde o início do fenômeno, para que a experiência não seja traumática, uma vez que a continuidade do fenômeno poderá ser uma realidade e será necessário que a relação seja acolhida para o estabelecimento de uma convivência adequada.

Com frequência, o fenômeno de ouvir vozes pode ser associado à ideia do “amigo imaginário”, comum no desenvolvimento infantojuvenil. Nesse caso, as crianças constroem narrativas complexas, de forma criativa e sociável. Em estudo transversal europeu, observou-se que, de 1.472 adultos, 41% tiveram um amigo imaginário na infância; desses, 69% estavam associados a ouvir vozes e 61% em ver e sentir, contudo não foi encontrada associação entre essa experiência e alucinações na fase adulta^(18)^, o que demonstra que ouvir vozes, por si só, não é um indicativo de agravos futuros.

Neste estudo, as crianças narraram com densidade as características das vozes, e alguns aspectos foram observados, como perceber se havia falta de controle sobre as vozes, confirmar se não havia um estímulo auditivo externo óbvio, observar se os ruídos ou vozes consistiam em um nome sendo chamado, ou se as vozes se dirigiam à criança. Existe uma parte dos ouvidores de vozes que consegue conviver de maneira positiva com essas experiências^(18)^, mas é um processo complexo a ser desenvolvido pela criança. O medo foi a emoção mais experimentada pelas narrativas do fenômeno, o que corrobora a literatura vigente^(12,16,18-20)^. Essas reações podem estar associadas às características das vozes, como em caso do sentido atribuído às vozes (intrusivas, controladoras e ameaçadoras)^(20,21)^ e o número de vozes. O ocorrido também foi visto em estudo com 52 participantes, sendo que 20% relataram ouvir uma voz, 53%, entre duas e quatro vozes, 10%, entre cinco e 10 vozes, 8%, entre 11 e 20 vozes, e 9%, mais de 20 vozes, sendo que o número foi associado às repercussões negativas, principalmente com comando de ações^(19)^.

Diante da história das vozes, sua compreensão sobrevém a partir do contexto de vida de cada interlocutor. As inúmeras situações e atravessamentos que estão relacionadas ao início, continuidade ou cessação da experiência perceberam que, ao narrar suas histórias, deu-se início a um processo individual, permitindo aos interlocutores olharem para si e suas memórias, além do reconhecimento dos distintos momentos em que as vozes apareceram e quais as associações com a vida diária. Esses aspectos contribuíram com a possibilidade de reencontrar suas histórias.

Alguns acontecimentos, como o envenenamento do cachorro de Naruto e estar na escola para Girassol, identificaram-se como situações que desencadearam vozes e visões. Todavia, não há como afirmar a existência de uma causa para o aparecimento das vozes na vida de todas as pessoas que ouvem ou até que possa ser a expressão e produção de subjetividade, mas é passível de acontecer com a mais tenra idade.

Como supracitado na introdução deste manuscrito, as vozes em crianças tendem a ser transitórias, como visto no relato de Girassol. Estima-se que aproximadamente três quartos dos jovens deixam de ouvir as vozes dentro dos primeiros cinco anos de seu início^(7)^. Estudo caso controle, com 145 casos (ouvidores de vozes) e 148 controles, indicou que ouvir vozes se manteve em 18,2% dos casos aos 6 anos, com redução para 6,2% aos 11 anos^(19)^, sendo decrescente com a idade. A literatura indica que as vozes podem desaparecer por um período e depois retornar, principalmente em caso de adversidades na infância^(12)^, mas isso não deve ser compreendido como um problema, desde que a criança aprenda a criar uma convivência tranquila com a experiência de audição.

Contudo, esse processo pode ser dificultado pela representação social e influência histórica dos rótulos da psiquiatria convencional diante do ouvir vozes, ainda permeado por estigmas^(21,22)^, pela patologização da infância. Investigação qualitativa, conduzida com adolescentes nos Estados Unidos, demonstrou que esses estão cientes dos estigmas associados a ouvir vozes, sendo uma fonte de sofrimento. O estigma é, em sua prevalência, associado à exclusão por ser “diferente”, e essa rotulação tende a fazer com que a criança veja a voz de forma negativa, com reações emocionais diversas diante da vivência, não buscando serviços de saúde para o apoio, pelo medo da estigmatização profissional^(7)^.

O medo do estigma dos adolescentes por profissionais de saúde deve ser considerado, pois muitos profissionais ainda atuam em uma lógica biomédica e reducionista. Estudo conduzido com médicos^(23)^ demonstrou que esses representam o fenômeno de ouvir vozes na infância como um diagnóstico psiquiátrico, com estigma e relutância no cuidado. Fato a ser considerado é que essa visão foi vista em médicos da atenção primária e secundária, locais que deveriam ser o centro ordenador do cuidado a esse adolescente^(9)^, principalmente na promoção da saúde. O mesmo é visto em metassíntese conduzida com estudos sobre as percepções dos enfermeiros, que também demonstraram medo da agressão e, consequentemente, afastamento^(22)^. Sabe-se que essa perspectiva pode ser iniciada desde a graduação do profissional, sendo de vital importância a inclusão da temática na sua formação^(24)^.

As visões supracitadas repercutem na assistência. Estudo transversal^(3)^, realizado com 95 crianças que ouviam vozes, demonstrou que: 52,6% tinham mais de um diagnóstico psiquiátrico e que 38,9% tinham um diagnóstico psiquiátrico, demonstrando o fenômeno da patologização da infância; 11,6% faziam uso de medicações antipsicóticas, o que pode demonstrar o fenômeno de medicalização da infância; e apenas 19% foram indicados para algum grupo de psicoterapia, sendo que esta, em uma linha de cuidado, seria a abordagem mais potencializadora, como realizado pelo grupo “Crianças Unidas”.

Outro ponto é que o estigma também segue as famílias, influenciadas pela visão comunitária do fenômeno. Estudo conduzido com familiares notou que eles interpretavam a experiência do filho como “perigosa” e “assustadora”, associando-a a diagnósticos psiquiátricos e atuando com uma superproteção da criança^(6)^, fato prejudicial ao seu desenvolvimento pleno. Ponto a ser ressaltado é que este estudo também atuou com adolescentes, que referiram não se sentir à vontade para falar da experiência devido às visões das famílias^(6,25)^. Nesse caminhar, grupos familiares são necessários nos equipamentos da Rede de Atenção Psicossocial (RAPS), para possibilitar um acolhimento familiar e, consequentemente, a promoção do bem-estar da criança.

Quando uma criança comunica a alguém que está ouvindo vozes, ela pode estar buscando uma fonte de apoio e conforto para ajudá-la a conviver com a experiência^(26)^, sendo necessário superar o estigma e construir um espaço acolhedor às suas demandas^(27)^. Apesar de o grupo “Crianças Unidas” não ser o enfoque deste estudo, é válido ressaltar a sua potencialidade como espaço de cuidado. As interlocutoras refletiram sobre muitas questões a respeito de suas experiências, e o suporte do grupo oferecia estratégias sobre como lidar com as mesmas. Assim, foi possível que a criança, em contato com suas experiências subjetivas, experimentasse *insights* para lidar com as emoções presentes e, por conseguinte, com a própria vida. Como mencionado anteriormente, as crianças tendem a omitir a escuta de vozes devido à sua representação no contexto social; para isso, é necessário tempo para que a criança crie vínculo e perceba a segurança de narrar o fenômeno e se tornar protagonista dessa história, e o grupo demonstrou potencial para isso. Para estudos futuros, cabe refletir quanto sua aplicabilidade nos equipamentos da RAPS.

Diversos autores^(7,13,19,21)^ referem que quando uma criança assume que é ouvidora de vozes, ela pode se considerar apenas uma maneira diferente de vivenciar o mundo ao seu redor; é como uma variação humana, pois, assim como há destros e canhotos, há pessoas que têm experiência com vozes, visões, cheiros e outras sensações^(13,28)^. Destaca-se a importância de que as crianças possam descobrir o que de fato ajuda neste processo de estabelecer contato com a experiência^(6,12,28)^, pois leva tempo para que as pessoas aceitem que são ouvidores de vozes^(13)^. É necessário que as narrativas individuais da natureza, característica e história sejam vistas e reproduzidas na prática clínica e em outros estudos, caminhando em busca da ressignificação do fenômeno.

### Limitações do estudo

Este estudo apresenta como limitações: o número de participantes, contudo são narrativas ricas de crianças que participaram de um considerável número de encontros; a abordagem de coleta de dados online, considerando que muitas crianças podem se sentir retraídas para expor suas percepções, principalmente sob supervisão da família, e o alcance de crianças ser privado às que possuíam aparelho celular e internet; e a produção de dados ter se centralizado em dois estados brasileiros (Rio Grande do Sul e Minas Gerais). Para estudos futuros, recomenda-se ampliar a coleta de experiências, considerando que a cultura é um determinante de saúde.

### Contribuições para as áreas da saúde, enfermagem ou políticas públicas

As narrativas deste estudo são de vital importância para o avançar da literatura sobre a escuta de vozes em crianças, demonstrando a natureza, características e histórias que podem guiar a atuação de profissionais de saúde na construção de projetos terapêuticos singulares. Ainda, este estudo vai contra referenciais convencionais e biomédicos, transitando da lógica da patologização da infância para o momento da ressignificação da escuta das vozes, dando sentido às vivências e espaço para as narrativas das crianças.

## CONSIDERAÇÕES FINAIS

Este estudo buscou compreender as experiências de crianças que ouvem vozes por meio de suas narrativas, e a partir dessas, foi possível observar que as crianças experienciam as vozes, visões, cheiros e sensações de forma única, pessoal e intransferível. Diante da natureza das experiências, notou-se que todas as crianças possuíam vozes e nem todas tinham visões ou outras sensações. As crianças indicaram quando iniciaram a ouvir vozes, especialmente em relação ao mês, turno do dia e atividades realizadas no momento. Ao indicar as características das vozes, foi possível observar a descrição do gênero, faixa etária, tom de voz, frequência e conteúdo das vozes/visões. Neste estudo, a construção do diálogo sobre as experiências de ouvir vozes promoveu acolhimento e até mesmo uma convivência positiva com o fenômeno. Acredita-se na potencialidade do grupo como espaço de interlocução com as crianças, uma vez que promover o protagonismo infantil significa cultivar novas formas de acolher a experiência com a escuta das vozes.

## Data Availability

Os dados da pesquisa estão disponíveis no corpo do artigo.
